# How to bridge the gap? European medical plants used for treating oral mucositis: on the search for evidence

**DOI:** 10.1007/s00432-020-03124-x

**Published:** 2020-01-18

**Authors:** Judith Buentzel, Christoph Bauer, Jens Buentzel

**Affiliations:** 1 Department of Haematology and Medical Oncology, University Medical Center Göttingen, Georg-August University, Göttingen, Germany; 2 Department of Nephrology and Hypertension, Center for Internal Medicine and Medical Clinic III, Klinikum Fulda, Fulda, Germany; 3 Department of Otolaryngology, Head Neck Surgery, Südharz Hospital, Nordhausen, Germany

**Keywords:** Traditional European medicine, Phytopharmacy, Oral mucositis, Evidence-based medicine

## Abstract

**Purpose:**

Oral mucositis is a common, painful side effect of cancer treatment—be it locoregional (e.g. irradiation) or systemic (e. g. chemotherapy). Phytotherapy is often used by patients to alleviate symptoms. However, knowledge on which medical plants are recommended by literature about Traditional European Medicine (TEM), their effect(s) on symptoms and their efficacy is severely lacking. Therefore, we developed a novel approach to assess traditional knowledge of herbals used in TEM and searched the online databases for studies reporting effects of these plants.

**Methods:**

At first, online research did not yield a satisfying number of studies (MESH terms: “mucositis” OR “stomatitis” AND “herbal” OR “herbal medicine”). Trials were labelled by the country conducting the study. In parallel, we compiled a list of 78 plants recommended for treating oral mucositis by screening 14 books on TEM. Then, a “hit list” of the plants most often mentioned was composed and used further for a second online investigation using the Latin plant designations as MESH term. Studies of both online searches were pooled for analysis.

**Results:**

There is a gap between traditional knowledge and trials investigating medical plants used by TEM. Overall, herbal remedies alleviate oral mucositis and especially, gingivitis well. There is good evidence for using *Matricaria recutita* L., *Salvia officinalis* L., *Calendula officinalis* L. and *Thymus* spp. L. for treating oral mucositis.

**Conclusion:**

Clinical trials investigating medical plants known in TEM are rare. However, following our research strategy, we could extrapolate four plants with good evidence for alleviating symptoms of oral mucositis and gingivitis.

## Introduction

Oral mucositis is a common, painful side effect of cancer treatment—be it locoregional (e.g. irradiation) or systemic (e. g. chemotherapy, EGFR-inhibitors). The symptom describes the inflammation of the enoral mucosa and is -if induced by radiotherapy-often associated with xerostomia, dysgeusia or dental problems (Rubenstein et al. 2004; Barrach et al. 2015; Kato et al. 2017; Crowder et al. 2018). Patients may present with erythematous or atrophic lesions of the oral mucosa and in more severe cases with ulcers. Especially, the latter is often accompanied with pain and an increase of risk for mucosal bleeding, infections and a higher hospitalization rate (Rubenstein et al. 2004).

The rate of patients using complementary alternative medicine (CAM) in addition to the therapy regime or to alleviate side effects of cancer treatment ranges from 40 to 90% (Molassiotis et al. 2005; Micke et al. 2009; Huebner et al. 2014; Wortmann et al. 2016). However, only around 50% of these patients inform their general practitioner and 35%, their treating oncologist about using CAM. Herbal medicine is one of the most commonly used CAM therapies, yet health practitioners seem to play a minor role in providing information. Patients usually use family, friends and media as main source of information (Molassiotis et al. 2005; Huebner et al. 2014).

Herbal medicine has a long history as medical plants and were mainly used in primary care up to the eighteenth century. The knowledge of most herbs dates back to antiquity recorded by the works of Hippocrates of Kos, Galen and Dioscorides (Leonti and Verpoorte 2017). In Europe, herbal medicinal products fall under the European Medicines Agency (EMA) Guideline on Quality of Herbal Medicinal Products (EMA/CPMP/QWP/2819/00). There are three different market authorizations a company selling herbals may apply for. While the first category comprises phytopharmacological agents for which pharmaceutical quality, safety and efficacy have to be demonstrated by preclinical and clinical studies, the other categories include (I) a well-established medical use of a plant or (II) a herb in use for more than 30 years (and more than 15 years in Europe) (Fürst and Zündorf 2015). In opposite to the traditional phytopharmacy practised in Europe till the beginning of the nineteenth century, rational phytopharmacy bases on the premise that a rational phytopharmaceutical has a proven effect. The phytopharmaceutical is composed of substances responsible for the actual effect and substances that may modify stability or bioavailability (Schulz and Hänsel 2013).

There is no clear-cut definition describing TEM. According to a survey amongst experts, the following traditional branches or methods (mostly based on the antique medical traditions) are usually counted as part of TEM: ancient Greek medicine (Hippocrates of Kos: dietetics, res non naturales; Galen: humourism, nosology), medieval (monastic) medicine (Hildegard of Bingen) or naturopathic methods deviated from ancient Greek medicine during the nineteenth century. TEM does (usually) not include homeopathy and approaches which fall into the more esoteric category (e. g. demonology, astrology, alchemy and magic) (Uehleke 2007; Micke and Büntzel 2013).

As mentioned above, patients commonly use CAM additionally to standard treatment and herbals play a major role (Molassiotis et al. 2005; Wortmann et al. 2016). Several plants like *Matricaria recutita* L., *Althaea officinalis* L., *Malva sylvestris* L. or Calendula officinalis are known to TEM for treating oral mucositis (Büntzel et al. 2019). However, the German guideline of supportive care in oncology does only mention the first, *Matricaria recutita* L., citing an insufficient level of evidence due to the lack of clinical trials (or low evidence) or due to studies not really launched to German oncology practitioners (Leitlinienprogramm Onkologie|S3-Leitlinie Supportive Therapie 2017). This astonishing discrepancy between plants reported by TEM (Büntzel et al. 2019) and the obvious lack of information available in the national guidelines is alarming—considering our patients’ use of herbal remedies (Molassiotis et al. 2005). This argues against treating herbal remedies as the red-headed stepchild of supportive cancer care and for (re)assessing which plants are commonly recommended by TEM and for investigating whether these are backed up by clinical trials.

Here we show a novel approach that may help to bridge the gap between TEM and the lack of evidence described above. We chose the oral mucositis as this is a common, easily assessed side effect of (systemic) oncological treatment.

## Methods

### Systematic literature research

Selection of databases: we selected PubMed as medical open access database and AGRICOLA as broader open scientific database. Furthermore, we tried to include CAM-specific data pools. The database of the University of Witten-Herdecke (“CAM-base”) yielded zero hits for oral mucositis. The last update was in 2010. The Veronica-Carstens-Stiftung offers additional data pool. Unfortunately, this database focusses on homeopathy alone.

**Fig. 1:**
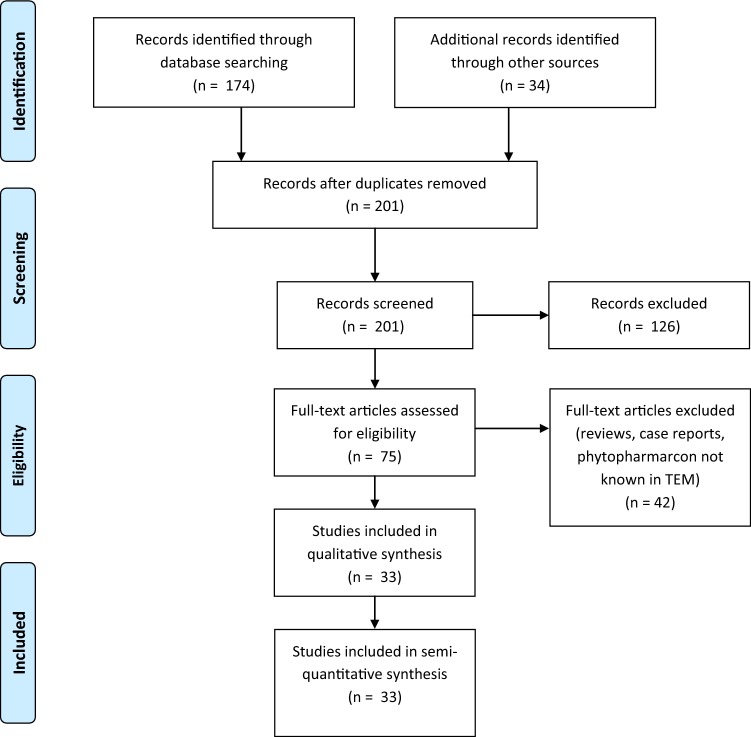
PRISMA flow diagram, first systematic literature research. Qualitative synthesis: Studies included investigating a plant for treating oral mucositis and describing an effect. Quantitative synthesis: Studies measuring or recording the effect, e.g., bleeding score gingivitis, and statistically comparing control/standard of care with the intervention group

We performed a systematic literature research using the online databases PubMed (174 hits) on 17th of February 2019 and AGRICOLA (83 hits, 4th of October 2019) to assess studies investigating herbal remedies for treating oral mucositis. The following MESH terms were used: “mucositis” OR “stomatitis” AND “herbal” OR “herbal medicine”. Articles were screened for studies using medical plants (historically) known to TEM. We applied no language restrictions or filters to the search, however only abstracts written in English were included into the final screening of literature. Abstracts identified by literature search were independently screened by two investigators (Ju. B. and J. B.). Abstracts were screened for the following inclusion criteria: (I) phytopharmacon is (historically) known to TEM; (II) used in clinical trials; (III) information available in English. Reference lists of the articles meeting the inclusion criteria were then screened via hand search to identify additional studies (34 hits). In summary, we were able to identify 33 studies suitable for further analysis (Fig. 1, Table 1). These were assessed for (I) plants reported for treating oral mucositis and (II) the country performing the research.

**Table 1:** Studies investigating herbal treatments for oral mucositis, first online search on PubMed and AGRICOLA

Author	Year	Orign	Plants investigated	
Ghorbani et al. (2018)	2018	Iran	Camellia sinensis KUNTZE	
Moriyama et al. (2018)	2018	Japan	*Glycyrrhiza glabra* L. *Rheum* L. *Zingiber officinale* ROSCOE	
Nishikawa et al. (2018)	2018	Japan	*Zingiber officinale* ROSCOE	
Kato et al. (2017)	2017	Japan	*Zingiber officinale* ROSCOE *Matricaria recutita* L.	
Dos Reis et al. (2016)	2016	Brazil	*Matricaria recutita* L.	
Nasry et al. (2016)	2016	Egypt	*Glycyrrhiza glabra* L. Acacia nilotica. J.H. Hurter & Mabb	
Tavakoli Ardakani et al. (2016)	2016	Iran	*Matricaria recutita* L. *Mentha* × *piperita* L.	
Mutluay Yayla et al. (2016)	2016	Turkey	*Salvia officinalis* L. *Mentha* × *piperita* L. *Thymus serpyllum*	
Braga et al. (2015)	2015	Brazil	*Matricaria recutita* L.	
Haghpanah et al. (2015)	2015	Iran	*Zingiber officinale* ROSCOE	
Matsuda et al. (2015)	2015	Japan	*Zingiber officinale* ROSCOE	
Hatakeyama et al. (2015)	2015	Japan	*Zingiber officinale* ROSCOE	
Miranzadeh et al. (2015)	2015	Iran	Achillea millefolium L.	
Andishe Tadbir et al. (2015)	2015	Iran	Matricaria recutita L.	
Yamashita et al. (2015)	2015	Japan	*Zingiber officinale* ROSCOE	
Seyyedi et al. (2014)	2014	Iran	*Matricaria recutita* L.	
Gavanji et al. (2014)	2014	Iran	*Punica granatum* L	
Aoyama et al. (2014)	2014	Japan	*Zingiber officinale* ROSCOE	
Ahmed (2013)	2013	Iraq	*Olea europaea* L.	
Ahmed et al. (2013)	2013	Iraq	*Olea europaea* L.	
Ghalayani et al. (2013)	2013	Iran	*Punica granatum* L.	
Abdollahzadeh et al. (2011)	2011	Iran	*Punica granatum* L.	
Das et al. (2011)	2011	India	*Glycyrrhiza glabra* L.	
Babaee et al. (2010)	2010	Iran	*Myrtus communis* L.	
Kono et al. (2010)	2010	Japan	*Zingiber officinale* ROSCOE	
Puataweepong et al. (2009)	2010	Thailand	*Aloe vera* L.	
Shabanloei et al. (2009)	2009	Iran	*Matricaria recutita* L.	
Martin et al. (2008)	2008	USA	*Glycyrrhiza glabra* L.	
Tiemann et al. (2007)	2007	Europe	*Matricaria recutita* L. Commiphora myrrha (NEES) ENGL Krameria lappacea (DOMBEY) BURDET & B.B. SIMPSON	
(Hubbert et al. (2006)	2006	Europe	*Salvia officinalis* L.	
(Shrivastava and John (2006)	2006	Europe	*Alchemilla vulgari*s L.	
(Su et al. (2004)	2004	USA	*Aloe vera* L.	
(Fidler et al. (1996)	1996	USA	*Matricaria recutita* L	

*L.* Linné

A second literature research was performed using 14 German books (listed in Table 2) on traditional medical plants and herbal remedies to assess, which plants were reported for treating mucositis/gingivitis or pharyngitis. We included books addressing different groups of interest: specialists (specialist literature, four books), laymen (popular science, five books) and users of wild growing edible or medical plants (plant identification books, five books). If a plant was reported to alleviate oral symptoms, we recorded it as known herbal remedy (Table 3). A resulting “hit list” of medical plants was generated by recording every report per plant and book screened. Each plant mentioned ≥ 6 times was included into a final “hit list” of the most common plants (Table 3).

**Table 2:** Popular science books screened for medical plants recommended for treating oral mucositis

References	
Achmüller, Arnold. 2012. *Teufelskraut, Bauchwehblüml, Wurmtod: das Kräuterwissen Südtirols : Mythologie, Volksmedizin und wissenschaftliche Erkenntnisse*. Bozen: Edition Raetia	PS
Adler, Martin. 2010. *Lehrbuch Naturheilverfahren: 106 Tabellen*. Stuttgart: Georg Thieme Verlag	SL
Hensel, Wolfgang. 2014. *Welche Heilpflanze ist das? : [über 350 Heilpflanzenarten aus ganz Europa kennenlernen und sicher bestimmen]*. Stuttgart: Kosmos	PI
Jänicke, Christof, Jörg Grünwald, and Thomas Brendler. 2003. *Handbuch Phytotherapie: Indikationen, Anwendungen, Wirksamkeit, Präparate*. Stuttgart: WVG, Wissenschaftliche Verlagsgesellschaft	SL
Landespflege, Bayerischer Landesverband f Gartenbau u, Eleonore Hohenberger, and Willi Votteler. 2017. *Gewürzkräuter und Heilpflanzen*. 7th ed. München: Obst- und Gartenbauverlag des Bayerischen Landesverbandes für Gartenbau und Landespflege e.V	PI
Mayer, Johannes Gottfried, Bernhard Uehleke, and Kilian Saum. 2013. *“Das” große Buch der Klosterheilkunde: neues Wissen über die Wirkung der Heilpflanzen ; vorbeugen, behandeln und heilen*. Zabert Sandmann	PS
Niederegger, Oswald, and Christoph Mayr. 2005. *Hausbuch der Südtiroler Heilkraeuter Gesundheit aus der Natur*. Bozen: Athesia	PS
Pahlow, Mannfried. 2004. *Das große Buch der Heilpflanzen: Gesund durch die Heilkräfte der Natur*. Hamburg: Nikol Verlagsges.mbH	PS
Prentner, Angelika. 2017. *Heilpflanzen der Traditionellen Europäischen Medizin: Wirkung und Anwendung nach häufigen Indikationen*. Berlin: Springer-Verlag	PI
Rätsch, Christian. 2014. *Heilpflanzen der Antike: Mythologie, Heilkunst und Anwendung*. Aarau: AT Verlag	PS
Schönfelder, Ingrid, and Peter Schönfelder. 2001. *Der Kosmos Heilpflanzenführer*. Stuttgart: Kosmos	PI
Seitz, Paul. n.d. *Heil- Und Gewürzpflanzen Aus Dem Eigenen Garten*. 13. Auflage. Bonn: aid Infodienst Ernähung, Landwirtschaft, Verbraucherschutz	PI
Steigerwald, Petra-Angela. 2015. *Phytotherapie pocket*. Grünwald: Boerm Bruckmeier	SL
Wichtl, Max, and Franz-Christian Czygan. 2002. *Teedrogen und Phytopharmaka: ein Handbuch für die Praxis auf wissenschaftlicher Grundlage*. Stuttgart: Wissenschaftliche Verlagsgesellschaft	SL

*PI* plant identification book/flora, *PS* popular science book, *SL* specialist literature/book

**Table 3:** List of all plants found during literature research, sorted by number of times mentioned in literature

Latin designation	*N* (mentioned)	Latin designation	*N *(mentioned)
***Matricaria recutita* L.**	**13**	*Tilia cordata* MILL. et platyphyllos SCOP	2
***Salvia officinali*s et sclarea L.**	**12**	*Ulmus minor* MILL	2
***Althaea officinalis* L.**	**11**	*Violar tricolor* L. et *Viola arvensis* Murray	2
***Malva sylvestri*s L. et *Malva neglecta* WALLER**	**11**	*Achillea millefolium* L.	1
***Calendula officinalis* L.**	**10**	*Ajuga reptans* L.	1
***Plantago lanceolata* L.**	**10**	*Alnus glutinosa* L	1
***Potentilla erecta* L.**	**10**	*Aloe barbadensis* L. (BURM. F.)	1
***Cetraria islandica* L.**	**9**	*Angelica archangelica* L.	1
***Arnica montana* L.**	**8**	*Anthyllis vulneraria* L	1
***Polygonum aviculare* L.**	**8**	*Betonicum officinalis* L	1
***Agrimonia eupatoria* L.**	**7**	*Carum carvi* L.	1
***Potentilla anserina* L.**	**7**	*Centaurium erythraea* RAFN	1
***Quercus robur* L.**	**7**	*Fragaria vesca* L.	1
**Rubus sect. Rubus**	**7**	*Gentiana lutea* L	1
***Thymus vulgaris* L.**	**7**	*Geum rivale* L.	1
***Tussilago farfara* L.**	**7**	*Gnaphalium uliginosum* L.	1
***Prunus spinosa* L.**	**6**	*Hieracium pilosella* L.	1
***Vaccinum myrtillus***	**6**	*Menyanthis trifoliata* L.	1
*Alchemilla vulgaris* L.	5	*Mespilus germanica* L.	1
*Commiphora myrrha* (NEES) ENGL	5	*Myrtus communis* L.	1
*Geum urbanum* L.	5	*Nasturtium officinale* R. BR. IN AIT	1
*Rubus ideaus* L.	5	*Orchis morio* L.	1
*Verbena officinalis* L.	5	*Origanum dictamnus* L.	1
*Chamaemelum nobie* L.	4	*Origanum majorana* L.	1
*Lamium album* L.	4	*Paeonia officinalis* L.	1
*Mentha arvensis* L. et *Mentha* × *piperita* L.	4	*Polygonum bistorta* L.	1
*Pimpinella saxifraga* L.	4	*Prunella vulgaris* L.	1
*Ribes nigrum* L.	4	Pulmonaria officinalis L.	1
*Syzygium aromaticum* L.	4	*Punica granatum* L.	1
*Juglans regia* L.	3	*Sambucus nigra* L.	1
*Rosa gallica* L. et *Rosa centifolia* L.	3	*Sanguisorba officinalis* L.	1
*Allium cepa* L.	2	*Sanicula europaea* L.	1
*Geranium robertianum* L.	2	*Sempervivum tectorum* L.	1
*Glechoma hederace*a L.	2	*Tamarix* spp. L.	1
*Glycyrrhiza glabra* L.	2	*Teurcrium scorodoni*a L.	1
*Hyssopus officinalis* L.	2	*Trifolium arvense* L.	1
*Ocimum basilicum* L.	2	*Usnea* sp. DILL. ex ADANS	1
*Origanum vulgare* L.	2	*Veroninca officinali*s L.	1
*Rheum palmatum* L.	2	*Zingiber officinale* ROSCOE	1

Bold plants mentioned ≥ 6 times, *L.* Linné

**Fig. 2:**
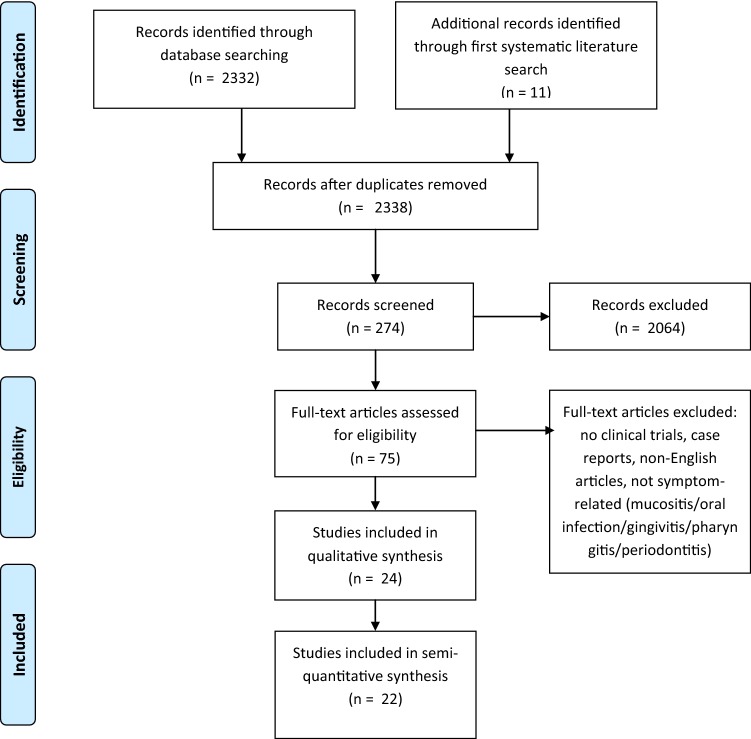
Synopsis, PRISMA flow diagram, second systematic literature research. Qualitative synthesis: Studies included investigating a plant for treating oral mucositis and describing an effect. Quantitative synthesis: Studies measuring or recording the effect, e.g. bleeding score gingivitis, and statistically comparing control/standard of care with the intervention group

We used these for a second systematic literature research on PubMed on the 10th March 2019 and on AGRICOLA on the 4th of October 2019. Latin designations of plants were used as MESH terms. We performed literature research separately for each plant. Reviews and in vitro or animal studies were excluded. Then, abstracts of clinical trials were screened for studies investigating “pain”, “inflammation” or “infection”. In a third step, we finally extracted studies focusing on “mucositis”, “stomatitis”, “gingivitis”, “periodontitis” or “pharyngitis” (for research strategy also refer to Fig. 2). Subsequently, we merged studies reporting clinical trials for plants of our “hit list” from our first research on PubMed and AGRICOLA with studies of our second systematic literature research. After removing duplicates we identified 24 clinical trials for further analysis (listed in Table 4). In summary, we developed a five step strategy to assess literature:

1. Initial use of online databases focusing on the symptom and plants chosen by the investigators for treatment.
2. Assessment of plants recommended by TEM literature referring to a balanced mixture of specialists’ and laymen’s literature. This step tries to represent, what plants have the highest probability to be used by our patients as the plants of our “hit list” are the ones which are most often recommended.
3. Merging the two first steps under the premise that the plants found in step (2) are the most commonly used and known plants for treating mucositis in Germany. Studies investigating these plants are included into further analysis.
4. Using Latin designations of plants of the “hit list” of step (2) as MESH terms on PubMed and AGRICOLA; manual screening and filtering for clinical trials investigating the symptom “oral mucositis”.
5. Studies extracted under (3) and (4) are now used for further analysis.

**Table 4:** Studies investigating one or several plants of the hit list

Authors	Year	*N* (pts)	Trial type (Jadad)	Level of evidence	Plants investigated	Plant compared to	Result
(Cabrera-Jaime et al. 2018)	2018	50	RCT* (4)	IB	*Plantago major* L.	Plant vs. control (chlorhexidine/bicarbonate wash)	No difference
(Charalambous et al. 2017)	2018	72	RCT (3)	IB	*Thymus vulgaris* L.	Plant vs. c control (saline wash)	Pos. plant
(Kato et al. 2017)	2017	14	CT (2)	IIIB	*Matricaria recutita* L., *Zingiber officinalis* ROSCOE (Hangeshashinto)	Plant	–
(Marucci et al. 2017)	2017	104	PCT* (5)	IB	*Matricaria recutita* L., *Calendula officinalis* L., *Aloe vera* L.,	Plant vs. placebo	No difference
(Azad et al. 2016)	2016	46	RCT (3)	IB	Cymbopogon flexuosus NEES EX STEUD., *Thymus zygis* L., *Rosmarinus officinalis* L	Plant vs. control (standard care)	Pos. plant
(Bardellini et al. 2016)	2016	87	RCT (3)	IB	*Matricaria recutita* L., *Calendula officinalis* L., *Aloe vera* L	Plant vs. control (hyaluronic acid)	Pos. plant
(Dos Reis et al. 2016)	2016	38	RCT (3)	IB	*Matricaria recutita* L.	Plant vs. control (ice cube)	No difference
(Goes et al. 2016)	2016	30	PCT* (5)	IB	*Matricaria recutita* L.	Plant vs. placebo	Pos. Plant
(Mutluay Yayla et al. 2016)	2016	60	RCT (3)	IB	*Salvia officinalis* L., *Mentha* × *piperita* L., *Thymus vulgaris* L.	Plant vs. basal oral care	Pos. plant
(Andishe Tadbir et al. 2015)	2015	43	PCT* (5)	IB	*Matricaria recutita* L.	Plant vs. placebo	Pos. plant
(Mahyari et al. 2016)	2015	60	PCT* (5)	IB	*Zingiber officinalis* ROSCOE, *Rosmarinus officinalis* L., *Thymus vulgaris* L.	Plant vs. placebo	Pos. plant
(Tavakoli Ardakani et al. 2016)	2015	60	PCT* (5)	IB	*Matricaria recutita* L., *Mentha* × *piperita* L.	Plants vs. placebo	Pos. plant
(Widén et al. 2015)	2015	32	PCT (4)	IIB	*Vaccinium myrtillus* L.	Plant vs. placebo (starch)	Pos. plant
(Braga et al. 2015)	2014	40	RCT (3)	IB	*Matricaria recutita* L.	Plant different concentrations vs. control (standard care)	Pos. plant
(Seyyedi et al. 2014)	2014	36	PCT* (5)	IB	*Matricaria recutita* L.	Plant vs placebo	Pos. plant
(Steinmann et al. 2012)	2012	20	CT (2)	IIB	*Salvia officinalis* L	Plant vs. Traumeel S ®	No difference
(George et al. 2009)	2009	30	RCT* (4)	IIB	*Matricaria recutita* L., *Salvia officinalis* L., *Commiphora myrrha* NEES ENGL., *Eucalyptus* L ‘HER	Plant vs. control (conventional dentifrice)	Pos. plant
(Schapowal et al. 2009)	2009	133	RCT* (4)	IB	*Salvia officinalis* L., *Ecchinacea* L.	Plant vs. control (chlorhexidine/ lidocaine)	No difference
(Shabanloei et al. 2009)	2009	83	PCT* (5)	IB	*Matricaria recutita* L.	Plant vs. placebo	Pos. plant
(Hubbert et al. 2006)	2006	286	PCT* (5)	IB	*Salvia officinalis* L.	Plant (different concentrations) vs. placebo	Pos. plant
(Tiemann et al. 2007)	2006	32	CT (2)	IIB	*Matricaria recutita* L., *Commiphora myrrha* NEES ENGL., *Krameria lappacea* (DOMBEY) BURDET & B. B. SIMPSON	Plant, baseline vs. termination time point	–
(González Begné et al. 2001)	2001	60	CT (2)	IIB	*Polygonum aviculare* L.	Plant (baseline vs. improvement over time)	Pos. plant
(Saller et al. 2001)	2001	145	RCT* (4)	IB	*Salvia officinalis* L., *Rheum* L	Plant/plant combination vs. control (acyclovir)	No difference
(Fidler et al. 1996)	1996	164	PCT* (5)	IB	*Matricaria recutita* L	Plant vs. placebo	No difference

Synthesis of first and second online literature research (PubMed)

*Blinded studies, *PCT *placebo-controlled trial, *RCT* randomized controlled trial, *CT *controlled trial, *L.* Linné

**Fig. 3:**
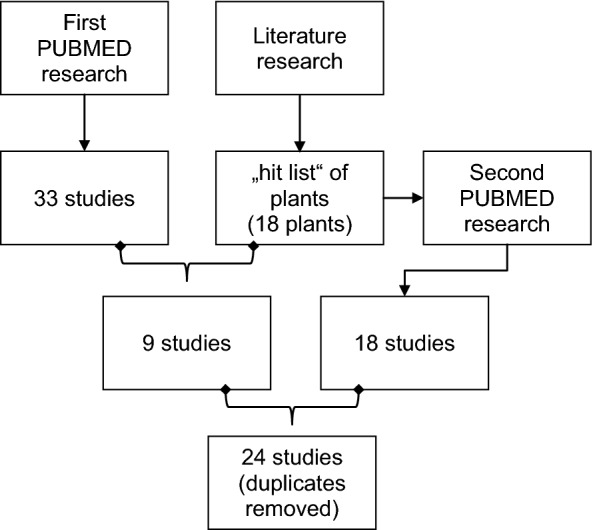
Overview over search strategy used for identifying plants and relevant studies included

A graphic overview of all steps of our research strategy is presented in Fig. 3.

### Qualitative analysis

The quality of studies that were later on included into semi-quantitative analysis, was assessed as following: The evidence level of the clinical trials included was evaluated according to the Oxford criteria (Oxford Centre for Evidence-based Medicine 2009). Trial quality was assessed according to the Jadad-Score. This validated score ranges from zero (very poor quality) to five (high quality) (Jadad et al. 1996, 2000).

### Assessment of the effect of medical plants on symptoms of oral mucositis

Studies extracted were sorted by symptoms as followed: mucositis/stomatitis, gingivitis/periodontitis and pharyngitis. Studies investigated the effect of “medical plant vs. placebo/ control remedy/baseline”. The p value describes the probability of error of the existing effect. We extracted *p* values for each medical plant compared to the control remedy. Afterwards, we used a simplified *p* value score for semi-quantitative analysis to describe the effect of herbal medicine on alleviating symptoms. The (*p* value based) score is shown in Table 5.

**Table 5:** Score based on the *p* value, used for describing the effect of medical plants compared to control remedy

Score (based on the *p* value)	Interpretation
0	No difference
1	Positive for plant (*p* < 0.05)
2	Strongly positive for plant (*p* < 0.01)

### Figures and illustrations

The free software “Inkscape” and/or Microsoft Office Powerpoint were used for generating all figures presented.

## Results

### Few studies investigate medical plants recommended for treating oral mucositis by TEM

The first search yielded 33 studies (Table 1). Here, *Matricaria recutita* L. and *Zingiber officinale* ROSCOE were the most investigated plants (nine studies each), followed by *Glycyrrhiza glabra* L. and *Punica granatum* L. (four studies). In summary, we compiled a list 17 different plants known to TEM reported by studies of our first systematic literature research. Next, we sorted studies according to the country performing the clinical trial. Astonishingly, only a small minority of studies (9.1%) was initiated in Europe. The majority (33.3%) was conducted in Iran. Japanese studies were mainly added, as these did also reported clinical trials on ginger—a plant already mentioned in the “De Materia Medica” of Dioscorides [first century AD, (Dioscorides and Berendes 2019)].

**Fig. 4:**
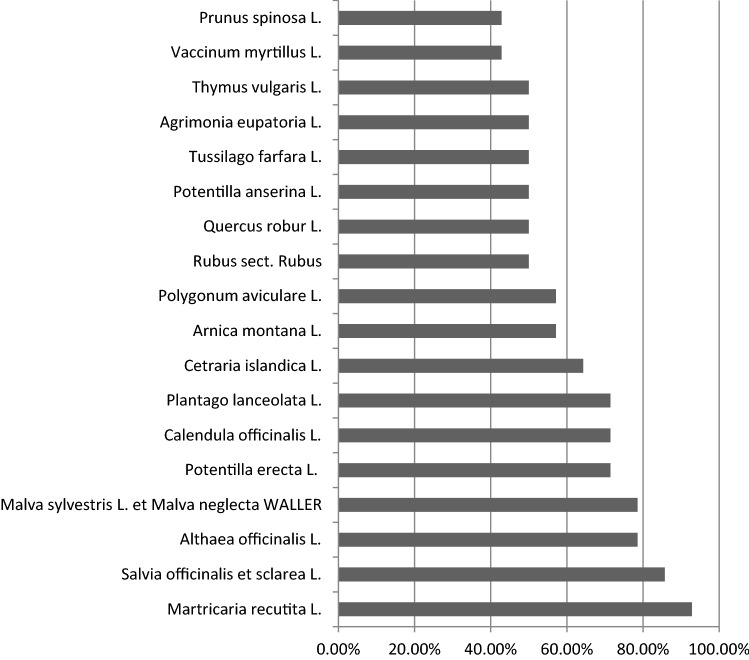
Hit-list of plants, overview over the 18 most commonly recommended herbal remedies. 100% = plant is mentioned in every herbal medicine book screened

To get an overview about TEM remedies, we conducted our second search using books on herbal remedies. Out of 14 books, we were able to list 78 herbs. Subsequently, we compiled a “hit list” of 18 plants that were recommended ≥ 6 times (Table 3, Fig. 4). Out of all plants *Matricaria recutita* L. (mentioned 13 times), *Salvia officinalis* et sclarea L. (mentioned 12 times), *Althaea officinalis* L. and *Malva sylvestris* L. et neglecta WALLER (mentioned 11 times) were the most often recommended medicinal herbs.

Afterwards, the overall list of plants found in our second literature research (books) was compared with plants found during our first systematic literature review. Finally, we found an overlap of 12 plants, meaning out of the 78 plants recommended by TEM only 15.3% of these were investigated by clinical trials. Furthermore, we found only clinical evidence for three medical plants out of the 18 herbal remedies of our “hit list” of most commonly recommended medical herbs.

In summary, we have an enormous gap/imbalance between medical plants recommended by TEM and clinical trials investigating these.

To overcome this imbalance we have performed a second online search as described above.

### Search results and characteristics of included studies

24 studies published between 1996 and 2018 meeting the inclusion criteria were included into the following analysis (Table 4). The number of patients participating in these clinical trials ranged from 14 to 286, we could include a total of 1725 patients. Out of all studies included four were controlled clinical trials, ten randomized clinical trials (RCT) and ten placebo randomized controlled trials, a total of 13 studies was blinded. The majority of studies had a high level of evidence (17/24 evidence level IB, refer to Table 4). 50.0% of studies were conducted in an oncological context (chemotherapy, radiotherapy or radio-chemotherapy). Studies investigated a total of 17 plants; out of these, seven herbs were part of our “hit list”. However, three herbs of the remaining plants investigated were mentioned by traditional TEM (*Aloe* L.: 1 hit; *Zingiber officinalis* ROSCOE: 1 hit; *Commiphora myrrha* NES.: 6 hits, refer also to Table 3).

### Results of semi-quantitative analysis on the overall impact of medical plants on treating oral mucositis

**Fig. 5:**
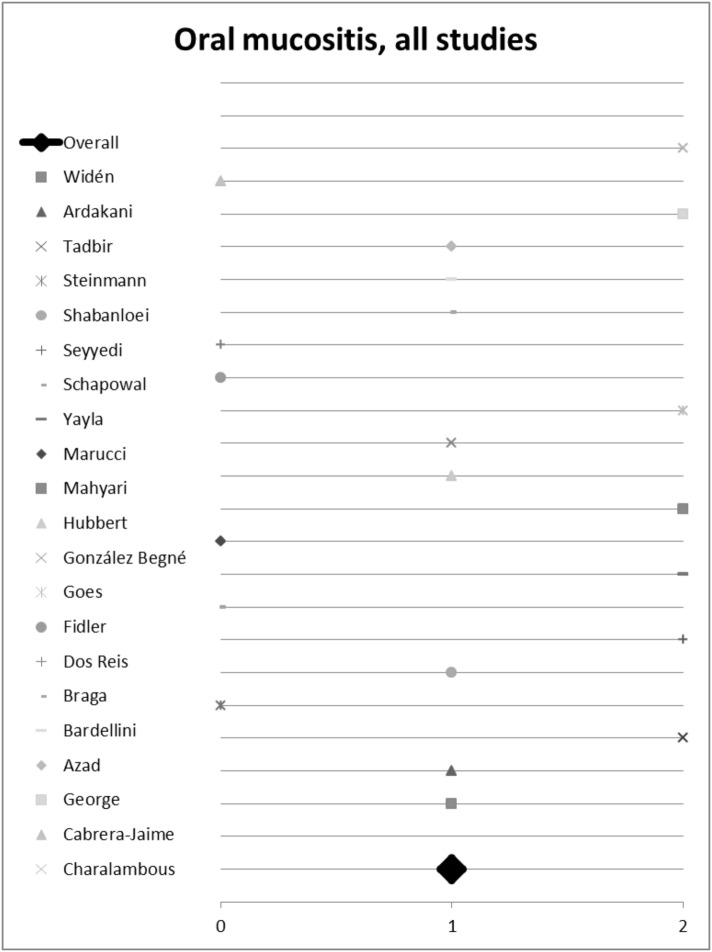
Semi-quantitative analysis on the overall impact of medical plants on treating oral mucositis using the semi-quantitative scoring system. A score ≥ 1 signifies a probability of error of < 5%. A score < 1 shows that no significant effect exists

22 of the 24 studies listed statistically compared the investigated plant with a control group and were thus included into semi-quantitative analysis (score). Seven studies found no difference between the effects of plants used versus the control. However, eight studies were positive and seven even highly positive for the effect of the plant investigated. Of the seven studies not being positive for herbal medicine, three studies compared the herbal remedy used to placebo, and four studies to conventional treatment (eg. chlorhexidine mouth wash (Cabrera-Jaime et al. 2018)) or ice cube (Dos Reis et al. 2016). In summary, there is evidence for using herbal remedies to alleviate oral mucositis (Fig. 5).

### Results of semi-quantitative analysis of the impact of medical plants on treating oral mucositis and stomatitis

**Fig. 6:**
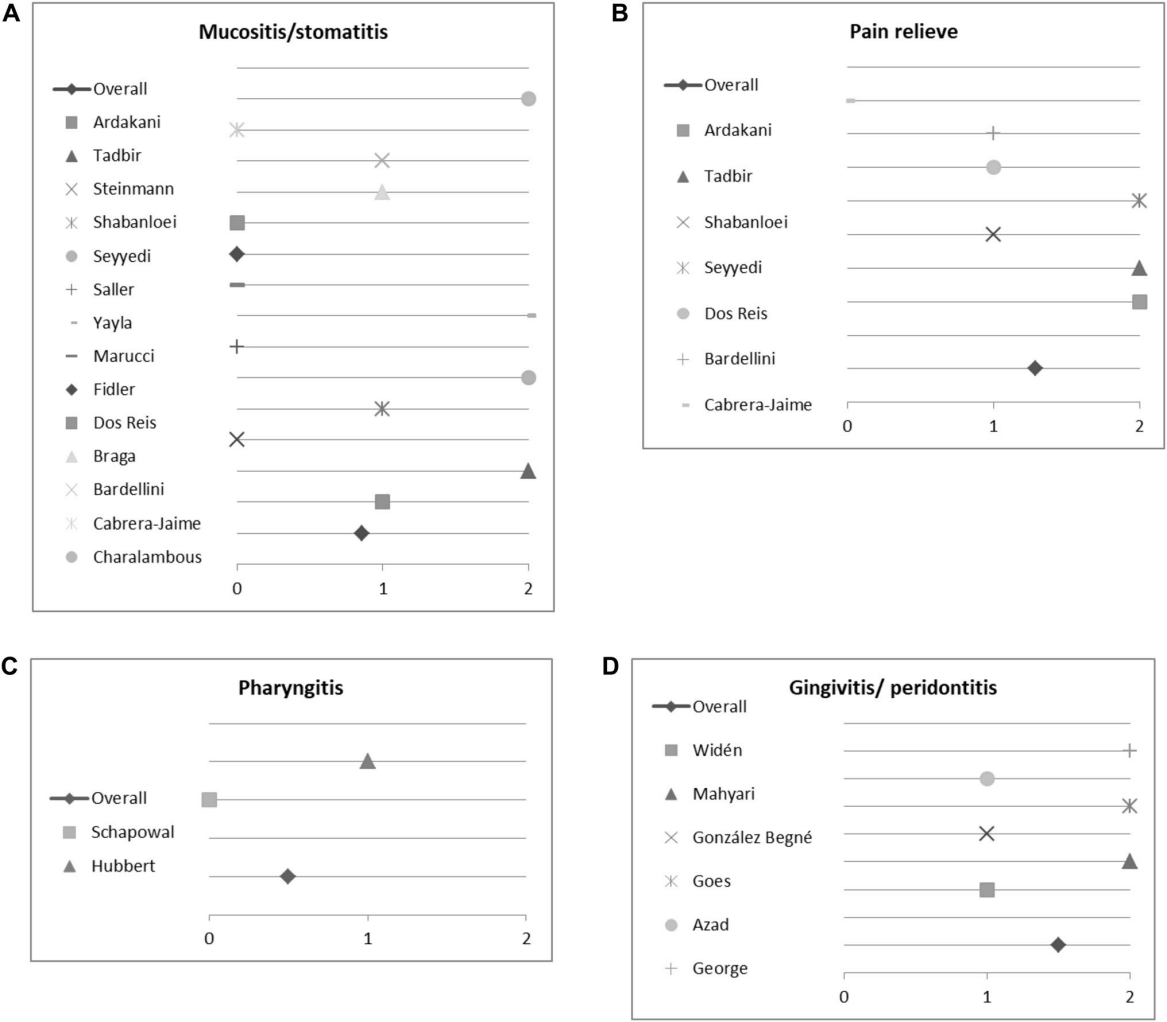
Impact of medical plants on treating oral mucositis and stomatitis, **a** semi-quantitative analysis of the effect on mucositis/stomatitis, **b** relieving pain, **c** pharyngitis and **d** gingivitis/periodontitis; semi-quantitative scoring system. A score ≥ 1 signifies a probability of error of < 5%. A score < 1 shows that no significant effect exists

16 studies investigated the impact of herbal medicine on mucositis (oral cavity) and stomatitis. However, only 14 quantified their results and were consequently used for semi-quantitative analysis. Six studies reported no difference between the plant investigated and control. It should be noted, that three of these studies compared the effect of the medical plant to standard of care/other remedies (Saller et al. 2001; Steinmann et al. 2012; Dos Reis et al. 2016). Notably three studies were highly positive for the medical plant investigated, while four showed a (weaker) positive effect (Fig. 6a). Taking all studies in account, we calculated an average score of 0.86. Conclusively, we found positive evidence for using medical plants. In following sub-analysis (Fig. 6b), we investigated studies also recording the effect on pain experienced during treatment of oral mucositis. Six out of seven studies reported that the herbal remedy investigated alleviated pain. Only Cabrera-Jaime et al. testing *Plantago major* L. for effects reported a negative outcome. The three studies reporting for a highly positive effect of the plant investigated compared *Matricaria recutita* L. against placebo. Other plants investigated were: *Aloe vera* L., *Calendula officinalis* L. and *Mentha* × *piperita* L. The overall score for alleviating pain was 1.29. Hence, we conclude that herbal medicine is quite effective for treating pain.

### Results of semi-quantitative analysis on the impact of medical plants on treating pharyngitis

Only two studies investigated medical plants, mainly *Salvia officinalis* L., for treating pharyngitis. One reported a positive outcome (Hubbert et al. 2006) while the other (Schapowal et al. 2009) described no difference to the control (Fig. 6c). However, the latter—Schapowal et al.—compared *Salvia officinalis* L. and Echinacea MOENCH to chlorhexidine/lidocaine spray, concluding that the combination of *Salvia officinalis* L. and Echinacea MOENCH was as effective as the standard of care. Taking this into account, we conclude that Salvia officinalis L. may be an effective alternative treatment to standard care.

### Results of semi-quantitative analysis on the impact of medical plants on treating gingivitis and periodontitis

Seven studies investigated the effect of medical plants on gingivitis or periodontitis, six were included for semi-quantitative analysis. All reported a positive or even strongly positive effect of the plant(s) used (Fig. 6d). Studies used bleeding on probing and/or gingival index and/or plaque index for assessing the symptom. The following plants were investigated: *Calendula officinalis* L., *Cymbopogon flexuosus* NEES EX STEUD., *Eucalyptus* L’HER, *Matricaria recutita* L., *Commiphora myrrha* (NEES) ENGL., *Polygonum aviculare* L., *Rosmarinus officinalis* L., *Salvia officinalis* L., *Thymus zygs* L., *Vaccinium myrtillus* L. and *Zingiber officnale* ROSCOE. The overall core was 1.5, indicating good evidence for treating gingivitis or periodontitis with herbal remedies mentioned above.

### Results of sub-group analyses for the evidence of single plants for treating oral mucositis

**Fig. 7:**
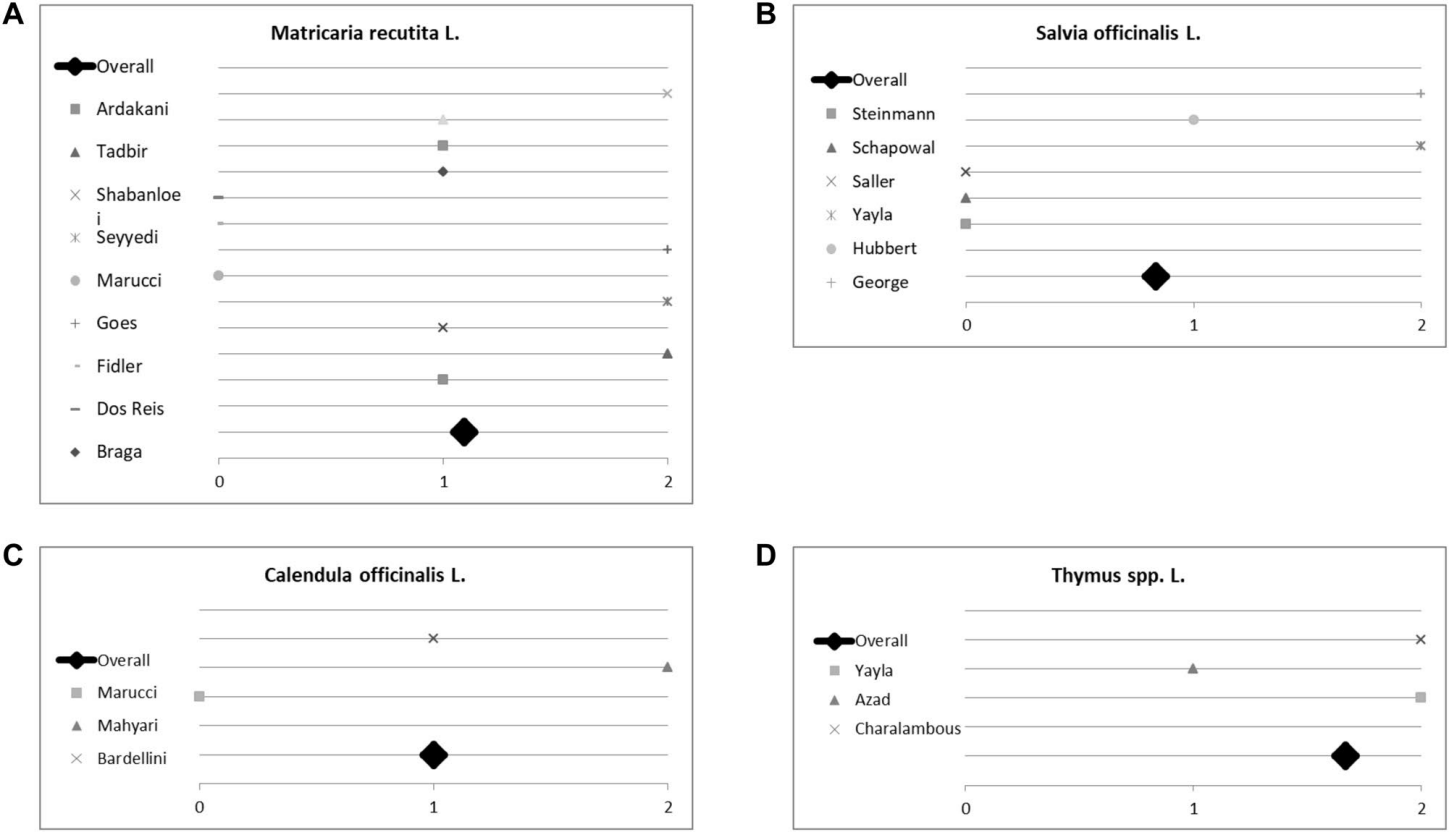
Impact of medical plants on treating oral mucositis, **a** semi-quantitative analysis of *Matricaria recutita* L., **b***Salvia officinalis* L., **c***Calendula officinalis* L. and **d***Thymus* spp. L.; semi-quantitative scoring system. A score ≥ 1 signifies a probability of error of < 5%. A score < 1 shows that no significant effect exists

**Fig. 8:**
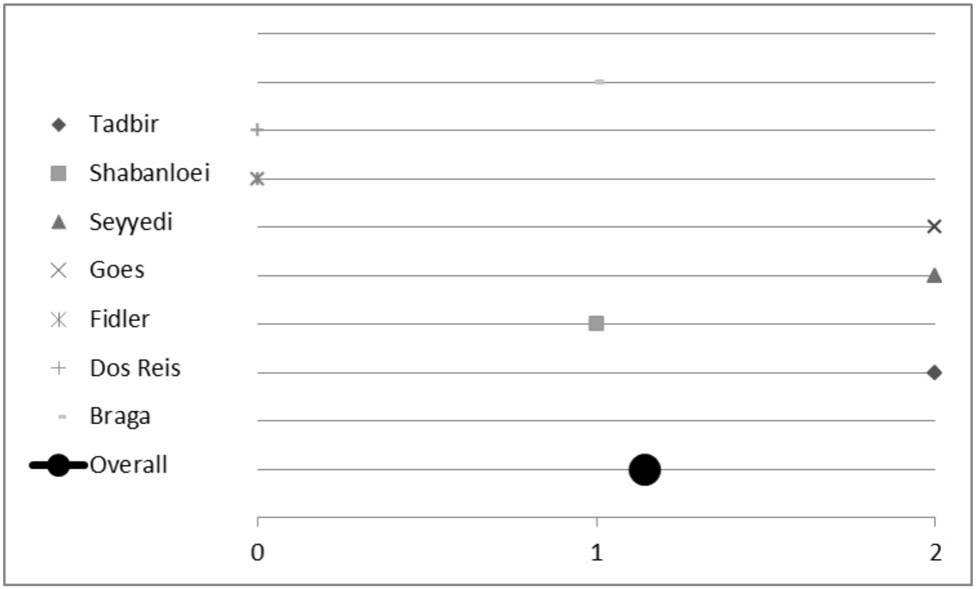
Impact of *Matricaria recutita* L. on treating oral mucositis, semi-quantitative analysis of studies only using chamomile; semi-quantitative scoring system. A score ≥ 1 signifies a probability of error of < 5%. A score < 1 shows that no significant effect exists

Seven plants included in this analysis were mentioned at least twice by different studies. Hence, we initiated a sub-group analysis to investigate their (individual) impact on treating mucositis. Plants and corresponding studies are listed in Table 4. Eleven studies investigated the effect of *Matricaria recutita* L.; three studies did not report any difference between the plant’s effect compared to placebo or control. However, *Matricaria recutita* L. was superior to placebo or control in the other eight studies included; the overall score for chamomile was 1.2 (Fig. 7a). Conclusively, treatment with chamomile seems to be a valuable and effective measure against oral mucositis. Out of these eleven studies, seven studies investigated the effect of *Matricaria recutita* L. alone without being combined with another medical plant. We used these for sub-group analysis. Here chamomile had an overall *p* value score of 1.1 (Fig. 8), indicating the plant is indeed effective. All studies had an evidence level IB and show a consistent, favourable effect of *Matricaria recutita* L., resulting in a Grade A recommendation, Oxford Criteria (Oxford Centre for Evidence-based Medicine 2009).

*Salvia officinalis* L. was investigated by six studies of which three did not report a difference between control and herbal remedy. Yet, the studies of Steinmann et al., Schapowal et al. and Saller et al. compared *Salvia officinalis* to Traumeel S, chlorhexidine/lidocaine and acyclovir respectively. All authors described Salvia officinalis as effective as the drug tested against. It should be taken into consideration that Saller et al. reported that a mixture of *Salvia officinalis* L. and *Rheum* L. was more effective than the application of *Salvia officinalis* L. alone (Saller et al. 2001). In summary, despite a lower score of 0.8 (Fig. 7b), we found evidence for using *Salvia officinalis* L. to treat oral mucositis. Due to George et al. being only a level IIB study and using a combination of herbs, we extrapolated data and were only able to give a Grade C recommendation, Oxford Criteria (Oxford Centre for Evidence-based Medicine 2009)).

*Calendula officinali*s L. was investigated by three studies; all used the plant in combination with other medical herbs. Marucci et al. did not detect any difference in grade III mucositis when testing Calendula officinalis against placebo. However, Mahyari et al. and Bardellini et al. reported a positive effect on gingivitis and stomatitis respectively. The overall score was 1.0 (Fig. 7c). There is some evidence for using *Calendula officinalis* L. (Grade B recommendation, Oxford Criteria]) for treating oral mucositis, but the small number of studies included should be taken into account.

We summarized studies investigating thyme under *Thymus* spp. L. as trials used two different sub-species of thyme (*Thymus vulgaris* L., *Thymus zygis* L.). All studies showed a positive effect of the plant, two were even highly positive (*p* value < 0.01). Studies were compared against controls. The overall score was 1.7 (Fig. 7d). Despite the small study number, we found good evidence for using *Thymus* spp. L. for treating oral mucositis as all studies consistently demonstrated the favourable effect of this herbal remedy, Grade B recommendation, Oxford Criteria (Oxford Centre for Evidence-based Medicine 2009).

*Aloe vera* L., *Mentha* × *piperita* L. and *Rosmarinus officinalis* L. were investigated in combination with other plants by two studies each showing scores of 0.5, 1.5 and 1.5 respectively. Due to the small study number, we hesitate to judge the role these plants might play for treating oral mucositis. Plants investigated by ≤ 2 trials are also listed in Table 6.

**Table 6:** Plants most often investigated, studies sorted by author and year

*Matricaria recutita* L.		*Salvia officinalis* L.		*Calendula officinallis* L.		*Thymus* spp. L	
Studies investigating herbs for treating oral mucositis (scored)							
Bardellini*	2016	Mutluay Yayla*	2016	Marucci*	2017	Charalambous	2018
Dos Reis	2016	Steinmann	2012	Bardellini*	2016	Azad*	2016
Goes	2016	George*	2009	Mahyari*	2015	Yayla*	2016
Tavakoli Ardakani*	2015	Schapowal*	2009				
Marucci*	2015	Hubbert	2006				
Anidshe Tadbir	2015	Saller*	2002				
Braga	2014						
Seyyedi	2014						
George*	2009						
Shabanloei	2009						
Fidler	1996						

*Aloe vera* L.		*Mentha* × *piperita* L.		*Rosmarinus officinali*s L.		*Cymbopogon flexuosus* SPRENG	
Further studies of medical plants (not scored)							
Marucci*	2017	Yayla*	2016	Azad*	2016	Azad*	2016
Bardellini*	2016	Ardakani*	2015	Mahyari*	2015		

Ecchinacea L.		*Eucalyptus* L’HER		*Commiphora myrrha* (NEES) ENGL		*Plantago major* L.	
Further studies of medical plants (not scored)							
Schapowal*	2009	George*	2009	George*	2009	Cabrera-Jaime	2018

*Polygonum aviculare* L.		*Rheum* L.		*Vaccinum myrtillius* L.		*Zingiber officinalis* ROSCOE	
Further studies of medical plants (not scored)							
González Begné	2001	Saller*	2002	Widén	2015	Mahyari*	2015

The upper part of the table lists a subject of scoring (refer also to Fig. 5, 6, 7 and 8)

*Plant was used in combination with other medical herbs

## Discussion

Oral mucositis is a common side effect of cancer treatment (Rubenstein et al. 2004; Barrach et al. 2015). More than half of our patients use CAM additionally to standard of care and here herbals play a major role (Molassiotis et al. 2005; Huebner et al. 2014). 39% of head neck cancer patients are searching for complimentary approaches. 25% of those are using medical herbs (Büntzel et al. 2018). However, information on the type and evidence of medical plants used by our patients is rare. Yet, popular science books on TEM regularly (and rightly) advise the user to ask the treating physician for advice [for example (Malm and Möbus 2018)]. This poses a problem -where to look at for reliable data? Our first online research revealed a total of 33 studies investigating the effect of medical plants on oral mucositis. Taken together, we did not get an abundant amount of studies; furthermore, the plants most often investigated (with the exception of *Matricaria recutita* L.)—*Zingiber officinale* (ginger) ROSCOE, *Glycyrrhiza glabra* L. (liquorice) and *Punica granatum* L. (pomegranate)—might not represent the herbals typically used or recommended in Germany. However, the composition of this list of plants is not that astonishing, considering that out of these 33 studies only three were initiated in Europe. Most of these trials were conducted by Iranian study groups. This overlap of both medical systems is understandable. The usage of similar plants is explained by shared history: Arabic culture translated, preserved and developed the Greek/Roman knowledge on medical plants, which was later transmitted back to medieval Europe e. g. via the first medical schools of Salerno or al-Andalus (Leonti and Verpoorte 2017).

But how to assess which plants are the most commonly recommended in (popular) science literature in Germany? We took a leap back and screened several phytopharmacy/TEM books available on the German market for herbal remedies against oral mucositis/gingivitis and were surprised by the huge variety of plants (78 plants) recommended. However, only 12 plants of these were tested by studies found during first online literature research. This first part of our review shows an imbalance between research and daily life of our patients. It also describes a gap between scientific knowledge and clinical real-life.

Therefore, we compiled a list of the most often mentioned herbs and initiated a second online literature research using Latin plant designations as MESH terms. This might restrict the output of studies, as some authors may use common names—e.g. chamomile instead of *Matricaria recutita* L.—but is necessary, as we appreciate the huge variety of possible designations for one plant- be it in German or in English. So we here present an innovative approach to (1) get an impression which plants are the most commonly recommended herbals by TEM (and thus might have the highest probability to be used by our patients) and to (2) maximize the study count for later (semi-) quantitative analysis.

Despite having found 24 studies during qualitative analysis, we decided against calculating hazard ratios for meta-analysis as it would have severely reduced the study number available. We chose a compromise developing the score based on the *p* value (refer to Table 5) for semi-quantitative analysis of the 22 studies finally included. Herbal remedies are suitable to alleviate oral mucositis. A positive effect is described for most plants, excluding *Plantago major* L. While study number is too small to assess the value of plants against pharyngitis, we could describe a positive effect of most herbs on oral mucositis and gingivitis. Overall, chamomile was the most often investigated plant and also the herb at the top of our “hit list” of plants. Here, we show that *Matricaria recutita* L. has a good effect on mucositis: Out of the seven studies investigating the influence of herbals on oral pain (Fig. 6b), four studies used *Matricaria recutita* L. (no combinations with other plants) and reported all a positive effect on alleviating symptoms. Therefore, we propose to revaluate the national guidelines of supportive care management in oncology, where chamomile is excluded from recommendation due to a low level of evidence/missing studies (Leitlinienprogramm Onkologie|S3-Leitlinie Supportive Therapie 2017). Other plants should also be reconsidered for treating oral mucositis: *Salvia officinalis* L. and *Thymus* spp. L. did show a positive effect on oral mucositis. Both plants contain essential oils that have been shown to have an antimicrobial activity against multidrug-resistant *S. aureus* (Tardugno et al. 2018; Kot et al. 2018).

Conclusively, we hereby present an approach to evaluate herbal plants used and offered by TEM and to assess the evidence for these recommendations of TEM. The role of herbals should be reassessed for evidence as they are commonly used by our patients (Molassiotis et al. 2005). It would be a step to improve physician’s counselling role. Furthermore it could be a step forward to higher quality of information platforms and websites as it was mentioned by Ciarlo et al (2018).
